# Calendar

**DOI:** 10.1111/iwj.13992

**Published:** 2022-10-28

## NORTH AMERICA

**10‐12 November 2022**

***AAWC 2022 Annual Conference***

**Salt Lake City, Utah, USA**

www.aawconline.org/2022annual-conference

**30 November ‐ 3 December 2022**

***Desert Foot Limb Salvage and Medical Surgical Podiatry Wound Care Conference***

**Phoenix, Arizona, USA**

www.go.event.com/1300979-0?pid=10008

## EUROPE

**13‐15 November 2022**

***Fourth Croatian Wound Association Symposium***

**Trogir, Croatia**

www.huzr.hr

**1‐2 December 2022**

***Fifth Nurnberg Congress (WUKO)***

**Nurnberg, Germany**

www.wuko2022.de

**4‐15 January 2023**

***International Conference on Wound Care and Hyperbaric Medicine (ICWCHM)***

**Zurich, Switzerland**

www.waset.org

**15‐17 January 2023**

***Journees Cicatrisations 2023***

**Paris, France**

www.cicatrisations2023.org

## INTERNATIONAL

**5 December 2022**

***International Conference on Wound Care, Tissue Repair and Regenerative Medicine***

**Dubai, United Arab Emirates**

www.conferenceindex.org/event/international-conference-on-wound-care-tissue-repair-and-regenerative-medicine-2022-December-Dubai-are

**7 December 2022**

***The Israeli Association for Incontinence, Stoma and Wounds (I.W.O.I.A) Conference***

**Tel Aviv, Israel**

www.facebook.com/groups/1618684384988994

**11‐12 January 2023**

***International Conference on Wound Care and Wound Management (ICWCWM)***

**Singapore, Singapore**

www.waste.org

**23‐24 February 2023**

***World Congress on Wound Healing and Critical Care***

**Dubai, United Arab Emirates**

www.woundcarecongress.alliedacadamies.com

